# TSpred: a robust prediction framework for TCR–epitope interactions using paired chain TCR sequence data

**DOI:** 10.1093/bioinformatics/btae472

**Published:** 2024-07-25

**Authors:** Ha Young Kim, Sungsik Kim, Woong-Yang Park, Dongsup Kim

**Affiliations:** Department of Bio and Brain Engineering, Korea Advanced Institute of Science and Technology, Daejeon 34141, South Korea; GENINUS Inc., Seoul 05836, South Korea; GENINUS Inc., Seoul 05836, South Korea; Samsung Genome Institute, Samsung Medical Center, Seoul 06351, South Korea; Department of Molecular Cell Biology, Sungkyunkwan University School of Medicine, Suwon 16419, South Korea; Department of Bio and Brain Engineering, Korea Advanced Institute of Science and Technology, Daejeon 34141, South Korea

## Abstract

**Motivation:**

Prediction of T-cell receptor (TCR)–epitope interactions is important for many applications in biomedical research, such as cancer immunotherapy and vaccine design. The prediction of TCR–epitope interactions remains challenging especially for novel epitopes, due to the scarcity of available data.

**Results:**

We propose TSpred, a new deep learning approach for the pan-specific prediction of TCR binding specificity based on paired chain TCR data. We develop a robust model that generalizes well to unseen epitopes by combining the predictive power of CNN and the attention mechanism. In particular, we design a reciprocal attention mechanism which focuses on extracting the patterns underlying TCR–epitope interactions. Upon a comprehensive evaluation of our model, we find that TSpred achieves state-of-the-art performances in both seen and unseen epitope specificity prediction tasks. Also, compared to other predictors, TSpred is more robust to bias related to peptide imbalance in the dataset. In addition, the reciprocal attention component of our model allows for model interpretability by capturing structurally important binding regions. Results indicate that TSpred is a robust and reliable method for the task of TCR–epitope binding prediction.

**Availability and implementation:**

Source code is available at https://github.com/ha01994/TSpred.

## 1 Introduction

T-cells are known to play a critical role in adaptive immune responses, detecting and eliminating infected cells in the body upon activation of T-cell receptors (TCRs). A TCR is activated when it binds to a peptide that is presented on the surface of an infected cell by the major histocompatibility complex (MHC). TCR sequences have an enormously large sequence diversity, which enables the recognition of a large number of different epitopes, thereby protecting the host from a wide variety of pathogens (Wooldridge *et al.* 2012). This sequence diversity is observed in the complementarity determining regions (CDRs) of the TCR. TCRs possess the property of cross-reactivity, such that the same TCR can bind to a number of different epitopes (Singh *et al.* 2017). At the same time, TCRs bind to epitopes in a highly specific manner, meaning that it is highly unlikely that a single TCR will bind to any randomly chosen epitope (Myronov *et al.* 2023).

Experimental approaches using pMHC multimers with bulk sequencing or single-cell sequencing (Zhang *et al.* 2018, Joglekar and Li 2021, Pai and Satpathy 2021) are used to detect TCR–epitope interacting pairs, but they are hindered by limitations in time and cost. This leads to a need for a reliable computational prediction method for determining TCR–epitope binding. However, the prediction of TCR–epitope binding is difficult, particularly for unseen epitopes, which are epitopes not seen during training (Grazioli *et al.* 2022, Deng *et al.* 2023). This is because the data available at the current moment is still too sparse compared to the huge sequence space of TCRs and epitopes (Weber *et al.* 2021). According to a study in 2023 (Hudson *et al.* 2023), public databases such as VDJdb (Bagaev *et al.* 2020), McPAS (Tickotsky *et al.* 2017), and IEDB (Vita *et al.* 2019) contain <1 million experimentally validated TCR–epitope pairs. Furthermore, there are only a few epitopes with a sufficient number of cognate TCRs, and a vast majority of epitopes have only one cognate TCR. While these factors pose challenges in the task of unseen epitope specificity prediction, it is an important subject that has various potential applications in the biomedical field. For example, a reliable unseen epitope prediction tool can be used for the prediction of immunogenic neoantigens, which has significant implications for cancer vaccine development (Waldman *et al.* 2020).

Many machine learning and deep learning-based methods have been developed to predict the interaction between TCRs and epitopes. Deep learning-based methods use a wide variety of architectures, such as convolutional neural networks (CNNs) (Montemurro *et al.* 2021, Moris *et al.* 2021, Montemurro *et al.* 2022, Jensen and Nielsen 2024), long short-term memory (LSTM) (Springer *et al.* 2020), autoencoder (Springer *et al.* 2020), and attention mechanism (Weber *et al.* 2021, Chen *et al.* 2023, Croce *et al.* 2024). Some tools, such as TITAN (Weber *et al.* 2021), ImRex (Moris *et al.* 2021), pMTnet (Lu *et al.* 2021), epiTCR (Pham *et al.* 2023), and TEINet (Jiang *et al.* 2023), consider only the information of the TCR beta chain. Other methods, such as ERGO (Springer *et al.* 2020), MixTCRpred (Croce *et al.* 2024), NetTCR-2.0 (Montemurro *et al.* 2021), NetTCR-2.1 (Montemurro *et al.* 2022), and NetTCR-2.2 (Jensen and Nielsen 2024), take into consideration the paired alpha and beta chain information. A recent benchmark study from the IMMREP 2022 workshop (Meysman *et al.* 2023) has reported that using paired chain data leads to more accurate predictions. Furthermore, some works (Montemurro *et al.* 2022, Croce *et al.* 2024, Jensen and Nielsen 2024) make a distinction between epitope-specific and pan-specific predictors. Epitope-specific predictors are specifically trained and tested for predicting the binding TCRs for the particular epitope, whereas pan-specific predictors can be applied to the prediction of binding TCRs for any given epitope. The authors of NetTCR-2.1 (Montemurro *et al.* 2022) and MixTCRpred (Croce *et al.* 2024) pointed out that models tend to perform worse when trained in a pan-specific manner compared to an epitope-specific manner. However, it is important for a model to be a reliable pan-specific predictor so that it can generalize well to unseen epitopes. Also, a recent study (Deng *et al.* 2023) investigated how the peptide imbalance in the dataset affects the performances of the currently developed models. The authors found that the models learn only on a few number of peptides that appear most frequently in the dataset. Consequently, as the peptide imbalance in the dataset increases, so does the extent of overestimation in model performances.

In this work, we present **T**-cell receptor binding **S**pecificity **pred**ictor (TSpred), a pan-specific approach for the prediction of TCR binding specificity using an ensemble of a CNN-based model and an attention-based model on paired chain TCR data. We leverage the predictive power of the CNN and the attention mechanism to build a robust model that can generalize well to unseen epitopes. In particular, we adopt a reciprocal attention mechanism which is specifically designed to capture the patterns underlying TCR–epitope binding. Based on a thorough evaluation of the model and comparison with other recent methods, we show that our model achieves state-of-the-art performances, in both seen epitope datasets and unseen epitope datasets. Also, based on an assessment of our model on a balanced dataset generated by down-sampling, we find that our model is most robust to bias caused by peptide imbalance in the dataset. Furthermore, we analyze the attention scores generated by the reciprocal attention layer and show that our model can capture the structurally interacting residue pairs that contribute to TCR–epitope binding.

## 2 Materials and methods

### 2.1 Negative sampling strategies

Two approaches for sampling negatives are widely used in this field: random shuffling and sampling from negative control data (Meysman *et al.* 2023). In the random shuffling approach, for each peptide-TCR pair, negative samples are generated by randomly sampling from TCRs binding to other peptides. This strategy must be conducted separately within the train and test partitions. Otherwise, a positive TCR in the training set can appear as a negative in the test set and vice versa, resulting in a significant degree of bias. Because of this, the random shuffling strategy can be difficult to use when there are only a few peptides with an imbalanced number of samples in a given data partition (see Supplementary Fig. S2 for a detailed explanation). Another limitation of the random shuffling strategy is that it has the potential to generate false negative pairs due to the cross-reactivity nature of the TCR–epitope interactions (Gao *et al.* 2023).

The second strategy is sampling from negative control TCR data without known epitope specificity, obtained from healthy individuals. A number of works (Moris *et al.* 2021, Grazioli *et al.* 2022, Dens *et al.* 2023, Meysman *et al.* 2023) have pointed out the problem with this approach, which is the bias arising from the difference in the positive and negative TCR sequence distributions. In some cases, this makes the classification problem too easy, resulting in exceedingly high model performances (Grazioli *et al.* 2022, Pham *et al.* 2023, Zhang *et al.* 2023). Due to this reason, a previous work (Moris *et al.* 2021) recommended using the random shuffling approach over sampling from negative control data. We carefully consider the limitations of each strategy and select the most appropriate strategy depending on the characteristics of each dataset.

### 2.2 Datasets

We conduct a comprehensive and rigorous evaluation of our model on a total of five datasets, two of which are provided by the authors of NetTCR-2.2 (Jensen and Nielsen 2024), and three of which are newly constructed (Table 1). First, the ‘NetTCR_full’ dataset, referred to as the ‘full dataset’ in NetTCR-2.2, consists of data from VDJDB (downloaded Aug. 2022) (Bagaev *et al.* 2020) and IEDB (downloaded Aug. 2022) (Vita *et al.* 2019), as well as a dataset from a 10x sequencing study (10x Genomics 2020, https://www.technologynetworks.com/immunology/application-notes/a-new-way-of-exploring-immunity-linking-highly-multiplexed-antigen-recognition-to-immune-repertoire-332554) for which iTRAP (Povlsen *et al.* 2023) has been used for denoising. The data are restricted to human and MHC class I data, and contain all three CDR sequences from both TCR α and β chains. The authors of NetTCR-2.2 performed a redundancy reduction step and excluded epitopes with <30 samples, which resulted in 6353 positive pairs across 26 peptides. Afterwards, the authors randomly split the 6353 positive pairs into five partitions and generated negative pairs in an 1:5 ratio by random shuffling within each partition. Whereas the nested 5-fold cross validation has been performed in NetTCR-2.2 (Jensen and Nielsen 2024), we use a modified nested 5-fold cross validation with only one inner loop and five outer loops to save computational time (Supplementary Fig. S1). This training strategy is used throughout this work.

**Table 1.** Summary of the datasets used for model training and evaluation in this study.

	NetTCR_full	IMMREP	NetTCR_bal	NetTCR_strict	Expanded_strict
Task	Prediction for seen epitopes			Prediction for unseen epitopes	
Training strategy	5-Fold cross validation (random split)			5-Fold cross validation (strict split)	
Data origin	‘Full dataset’ in NetTCR-2.2; derived from VDJdb, IEDB, 10x sequencing data (denoised)	IMMREP 2022 benchmark	NetTCR_full	NetTCR_full	NetTCR_full + additional pairs from VDJdb, McPAS, IEDB
Number of peptides	26	17	26	26	753
Number of positive samples	6353	1960	1893	6353	8908
Negative generation	Random shuffling (1:5)	Random shuffling (1:3) + negative controls (1:2)	Random shuffling (1:2)	Negative controls (1:1)	Random shuffling (1:5)

In addition, we use the ‘IMMREP’ dataset which has been generated from the IMMREP 2022 benchmark study (Meysman *et al.* 2023) and post-processed by the authors of NetTCR-2.2 (Jensen and Nielsen 2024). This dataset is also a 5-fold cross validation dataset with five randomly split partitions. The negative samples in this dataset have been generated by a combination of random shuffling and sampling from negative control data, with a final positive-to-negative ratio of 1:5. The negative control data come from the IMMREP benchmark study (Meysman *et al.* 2023) and consist of 15 957 TCR sequences with no known binding specificity obtained by 10x sequencing from 11 control individuals. This dataset consists of 17 different peptides.

Based on NetTCR_full dataset, we create another dataset called ‘NetTCR_bal’. NetTCR_bal is a balanced dataset which is created in order to minimize the impact of peptide imbalance on the model performance. It is generated by combining all positive pairs in the NetTCR_full dataset and down-sampling the data to 100 samples for each peptide. For the peptides with <100 positive samples, all samples are used. Again, all the positive pairs are randomly split into five partitions. Negatives are generated in a 1:2 ratio, which is the limit of the positive-to-negative ratio that can be achieved with the amount of data in each partition.

Furthermore, we construct the ‘NetTCR_strict’ dataset to evaluate our model on the unseen epitope specificity prediction task. We employ the ‘strict split’ method for creating this dataset, by randomly partitioning all peptides into five nonoverlapping folds and assigning associated pairs to each. Specifically, peptides appearing in one partition do not exist in any other partition. Since there are only a few peptides in each partition, results vary widely depending on how the epitopes are split. Thus, we split the data five times using five different random seeds, and conduct 5-fold cross validation for each data split. For the negative samples, we randomly sample from the previously mentioned negative control data from IMMREP benchmark (Meysman *et al.* 2023), with a positive-to-negative ratio of 1:1. We use negative controls here because each of the five test partitions generated by the strict split strategy contains such a small number of peptides with an imbalanced number of samples that it is not possible to achieve a 1:1 ratio by random shuffling in certain cases (Supplementary Fig. S2).

We also construct the ‘Expanded_strict’ dataset to assess our model on the task of predicting TCR specificity for unseen epitopes. We assemble data from VDJdb (downloaded September 2023) (Bagaev *et al.* 2020), McPAS (downloaded September 2023) (Tickotsky *et al.* 2017), and IEDB (downloaded November 2023) (Vita *et al.* 2019) databases and conduct pre-processing to obtain human, MHC class I, and paired chain data. This additional dataset is combined to the NetTCR_full dataset. The dataset is down-sampled to 500 samples per peptide, and the low frequency epitope exclusion is not performed. This yields a total of 753 peptides with 8908 positive samples. 5-Fold cross validation set is constructed using the strict split strategy. We generate negatives in each training, validation, and test partition via random shuffling in a 1:5 ratio. We construct this new ‘Expanded_strict’ dataset due to two reasons. First is that it is difficult to apply the random shuffling strategy to NetTCR_strict, due to the reasons described earlier. Second is that a relatively small number of epitopes (26) was used in NetTCR_strict, which may raise concerns regarding the generalization ability of the model to unseen epitopes.

### 2.3 Model architecture

**Figure 1.**
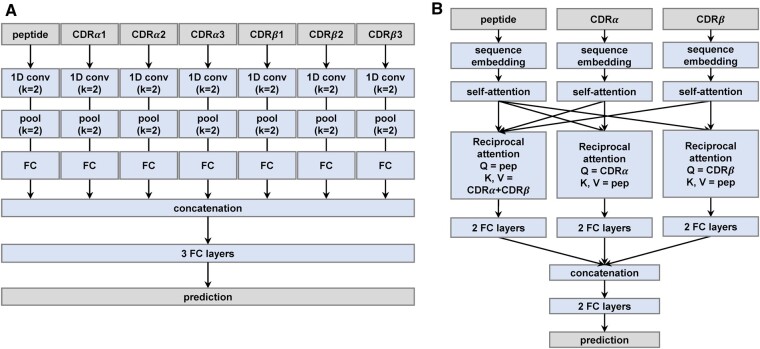
Overview of TSpred model architecture. (A) CNN-based model (TSpred_CNN). Each of the peptide and six CDRs pass through a 1D convolutional layer, pooling layer, and a fully connected (FC) layer. All vectors are concatenated and forwarded through a series of fully connected layers to output the final value. *k* = 2 refers to a kernel size of 2. (B) Attention-based model (TSpred_attention). In this model, the peptide, CDRα, and CDRβ sequences are the input. Each input is passed through a sequence embedding layer, self-attention layer, reciprocal attention layer, and two fully connected layers. All vectors are concatenated and forwarded through a series of fully connected layers to output the final value. The final TSpred model (TSpred_ensemble) is an ensemble model of (A) and (B).

In this study, we propose an ensemble model of two different models, a CNN-based model and an attention-based model (Fig. 1, Supplementary Note S1). In the CNN-based model (TSpred_CNN) (Fig. 1A), each of the peptide and the six CDR sequences are one-hot encoded and forwarded through a convolution module. The convolution module is composed of a 1D convolutional layer with a kernel size of 2, a max pooling layer with a kernel size of 2, and a fully connected layer. The outputs from each module are concatenated and passed through three fully connected layers with a sigmoid activation to produce the final output.

In the attention-based model (TSpred_attention) (Fig. 1B), the inputs are the peptide, CDRα, and CDRβ sequences. Here, the CDRα and CDRβ sequences are generated by zero-padding CDR1, CDR2, and CDR3 sequences to their maximum lengths and concatenating them together. Each input sequence is fed into a learnable embedding layer, followed by a multi-head self-attention layer. Then the vectors are each passed through a multi-head reciprocal attention layer, which takes the input sequence as the query and the other sequences as the key and value. Specifically, for the layer with the peptide as the query, the key and value are both the concatenated sequences of CDRα and CDRβ. For the layer with CDRα as the query, the key and value are both the peptide. The same applies to the layer with CDRβ as the query. This layer is designed so that the model can focus more on the relevant parts of the sequences of the interacting partners. After the reciprocal attention layer, each output is passed through two fully connected layers. The three resulting vectors are then concatenated, flattened, and fed into two fully connected layers with a sigmoid activation to produce the final output.

The final ensemble model (TSpred_ensemble) of the CNN- and attention-based models is obtained by taking the average of the predictions of each model. We use different training hyperparameters for the CNN- and attention-based models (Supplementary Note S1). The criterion for choosing the number of epochs is based on the ROC-AUC on the validation set.

### 2.4 Performance evaluation

For the model performance evaluation, we report the average of the model performances on the test set across the 5-folds. We use the metrics of Area Under the Receiver Operating Characteristic Curve (ROC-AUC) and Area Under the Precision-Recall Curve (PR-AUC). We also report the accuracy, precision, specificity, recall and *F*1-score based on a cutoff of 0.5. These metrics are calculated as follows:

$$\mathrm{Accuracy}=\frac{TP+TN}{TP+FN+TN+FP}$$

$$\mathrm{Precision}=\frac{TP}{TP+FP}$$

$$\mathrm{Specificity}=\frac{TN}{FP+TN}$$

$$\mathrm{Recall}=\frac{TP}{TP+FN}$$

$$F1=2\frac{\mathrm{Precision}\times \mathrm{Recall}}{\mathrm{Precision}+\mathrm{Recall}}$$

where TP, TN, FP, and FN are the number of True Positives, True Negatives, False Positives, and False Negatives, respectively.

### 2.5 Comparison to other methods

We compare our method with five other recent state-of-the-art methods, TITAN (Weber *et al.* 2021), epiTCR (Pham *et al.* 2023), TEINet (Jiang *et al.* 2023), MixTCRpred (Croce *et al.* 2024), and NetTCR-2.2 (Jensen and Nielsen 2024). TITAN takes the SMILES encoding of the peptide and full TCR β chain amino acid sequence as input, while epiTCR and TEINet take the peptide and CDRβ3 as input. MixTCRpred and NetTCR-2.2 take the peptide and all three CDRs from both α and β chains as input. TITAN is based on a convolutional attention mechanism, epiTCR is based on a random forest model, and TEINet is based on pre-trained encoders. MixTCRpred uses the transformer encoder architecture, and NetTCR-2.2 uses the convolutional neural networks. For TITAN, models are trained in the ‘pretrained semifrozen’ setting mentioned in the manuscript, and all three β chain CDRs are concatenated and provided as the TCR input. For both MixTCRpred and NetTCR-2.2, the pan-specific version of the model is used for assessment. MixTCRpred was re-implemented based on the model source code made available by the authors. We compare all models using the same training, validation, and test datasets.

## 3 Results

### 3.1 Prediction on seen epitopes

**Figure 2.**
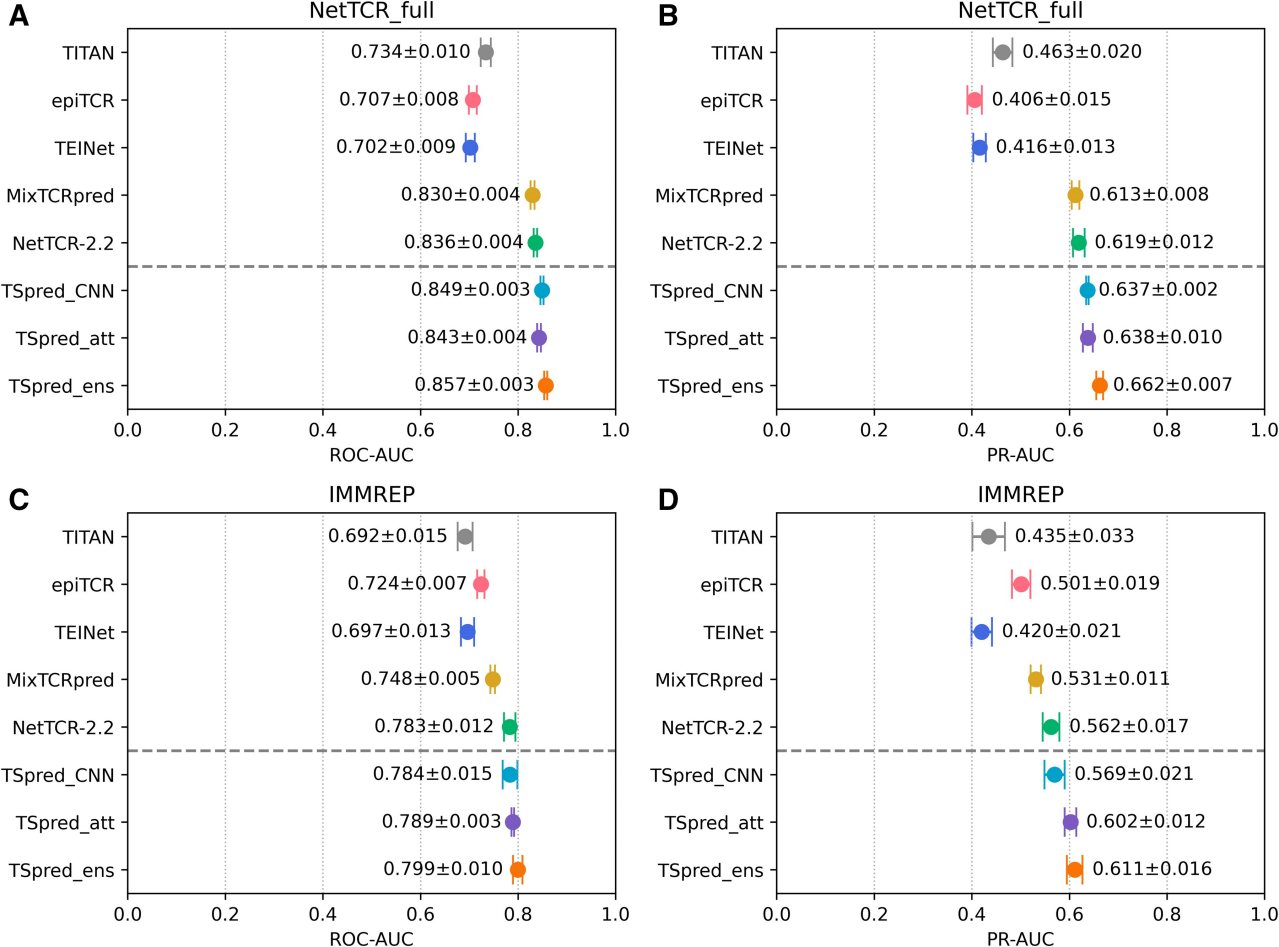
Performances of TSpred and other models on the seen epitope datasets (NetTCR_full, IMMREP). The colored dots represent the mean and the whiskers represent the standard deviation. (A) and (B) show the model performances in terms of ROC-AUC and PR-AUC on the NetTCR_full dataset, respectively. (C) and (D) show the model performances in terms of ROC-AUC and PR-AUC on the IMMREP dataset, respectively. The upper part of each subplot shows the results of the compared state-of-the-art methods, whereas the lower part of each subplot shows the results of the final TSpred model (TSpred_ensemble) and its individual components (TSpred_CNN and TSpred_attention). TSpred_att stands for TSpred_attention; TSpred_ens stands for TSpred_ensemble.

**Figure 3.**
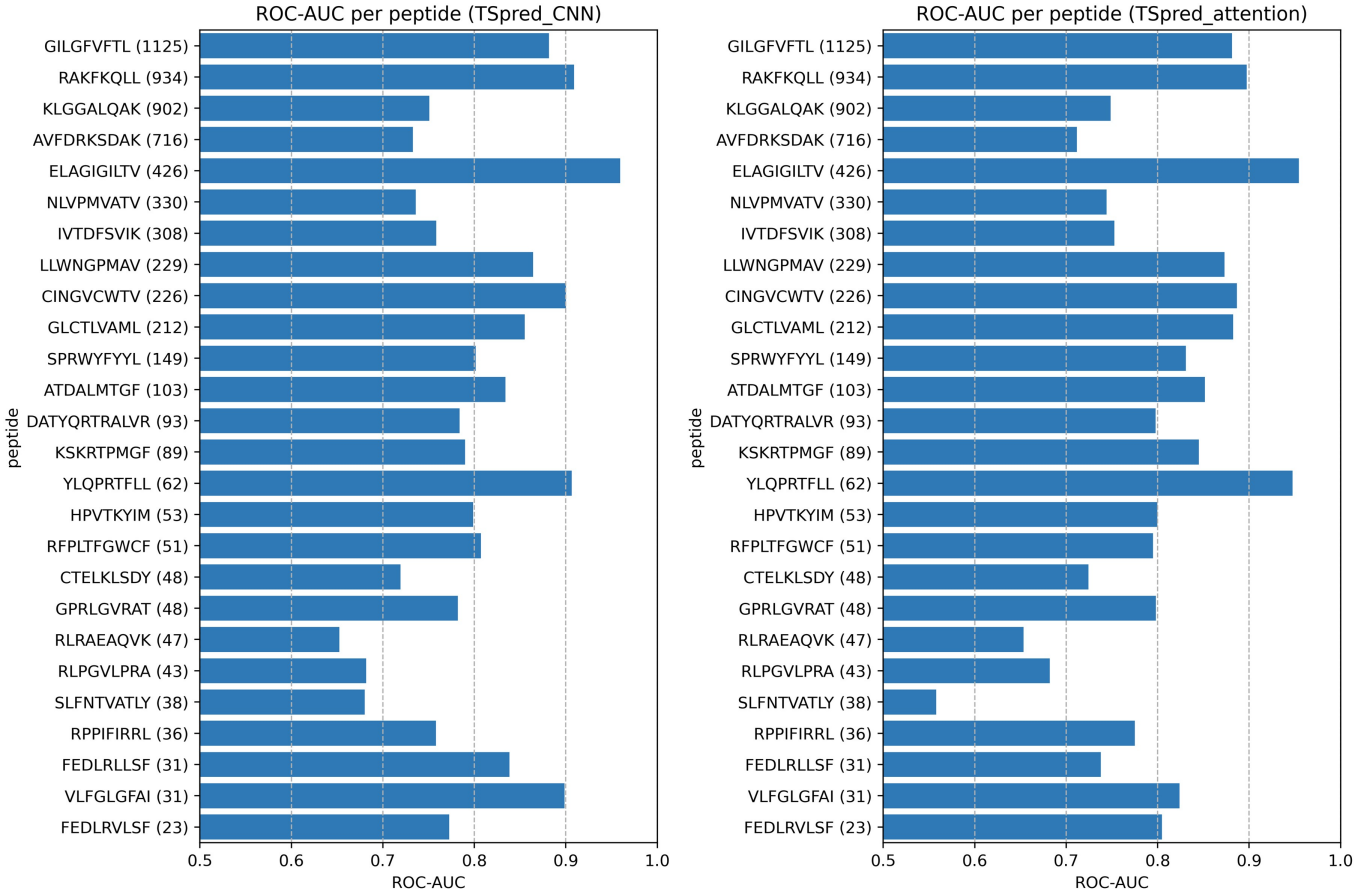
Per-peptide ROC-AUC performances of TSpred_CNN and TSpred_attention on the NetTCR_full dataset. The numbers shown in parentheses refer to the number of positive samples for each peptide.

We evaluate the performances of the final TSpred model (TSpred_ensemble) as well as the individual model components (TSpred_CNN and TSpred_attention) and other previously published methods on NetTCR_full dataset using ROC-AUC and PR-AUC (Fig. 2A and B). TSpred_CNN and TSpred_attention both achieve state-of-the-art results in terms of both metrics. By combining the predictive power of the two individual models, TSpred_ensemble achieves an even higher performance, with a mean ROC-AUC of 0.857 and a mean PR-AUC of 0.662. For this dataset, the methods using paired chain data outperform the methods using only beta chain data. Upon evaluation in terms of classification metrics, our model shows good performances in terms of accuracy, precision, specificity, and *F*1-score (Supplementary Fig. S3). We also inspect the relationship between the per-peptide ROC-AUC performances of TSpred_CNN and TSpred_attention and the number of positive samples corresponding to each peptide (Fig. 3). As expected, peptides with a higher number of samples tend to achieve higher ROC-AUC values. For TSpred_CNN, the five most frequent peptides show a mean ROC-AUC of 0.847, and the five least frequent peptides show a mean ROC-AUC of 0.789. Similarly, for TSpred_attention, the five most frequent peptides show a mean ROC-AUC of 0.839, while the five least frequent peptides show a mean ROC-AUC of 0.740.

Furthermore, we assess the performances of different methods on the IMMREP benchmark dataset for the task of predicting specificity for seen epitopes (Fig. 2C and D). Again, we observe that TSpred_CNN and TSpred_attention show better performances compared to the other tools, and that TSpred_ensemble even outperforms these two models, achieving a mean ROC-AUC of 0.799 and a mean PR-AUC of 0.611. We once again observe that using paired chain data as the model input leads to better prediction accuracy compared to using only beta chain data. In terms of classification metrics, our models show high accuracy, specificity, and *F*1-score (Supplementary Fig. S4).

### 3.2 Assessment on a balanced dataset

**Figure 4.**
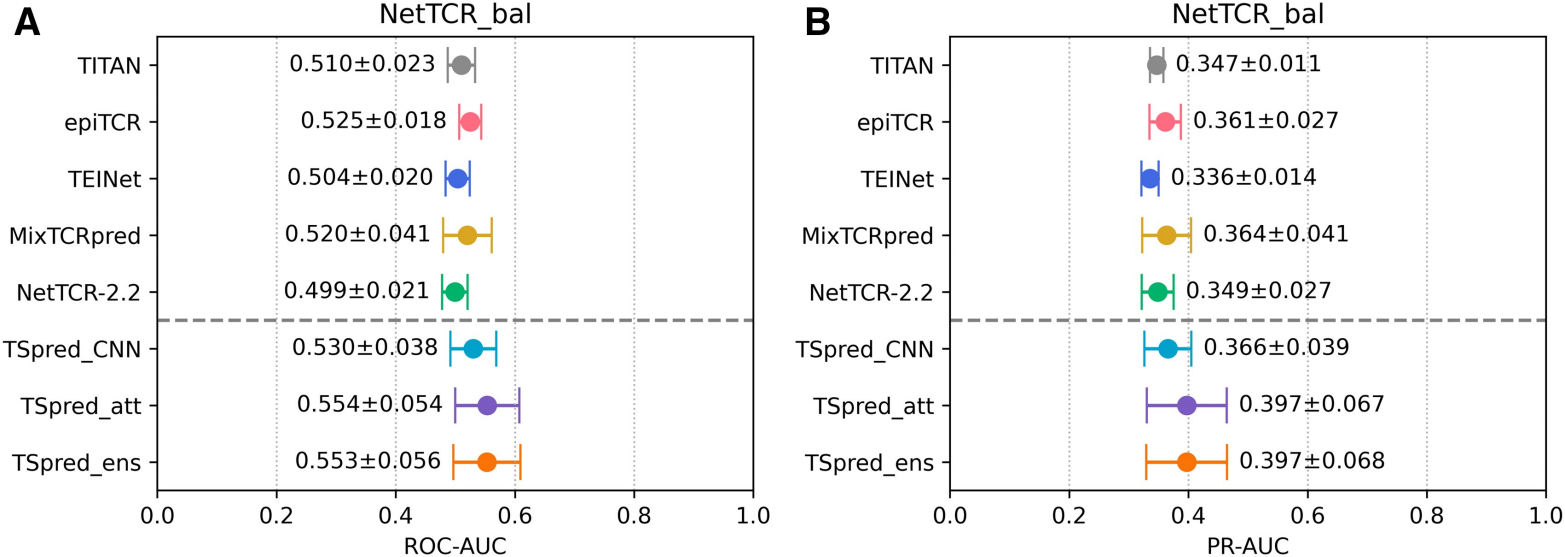
Performances of TSpred and other models on the NetTCR_bal dataset. The colored dots represent the mean and the whiskers represent the standard deviation. (A) and (B) show the model performances in terms of ROC-AUC and PR-AUC, respectively. The upper part of each subplot shows the results of the compared state-of-the-art methods, whereas the lower part of each subplot shows the results of the final TSpred model (TSpred_ensemble) and its individual components (TSpred_CNN and TSpred_attention). TSpred_att stands for TSpred_attention; TSpred_ens stands for TSpred_ensemble.

In order to rule out the bias caused by peptide imbalance on model performances, which has been pointed out by a recent study (Deng *et al.* 2023), we evaluate TSpred on a balanced dataset (NetTCR_bal) which is constructed by down-sampling the data from NetTCR_full dataset. We compare the performances of different methods in terms of ROC-AUC and PR-AUC (Fig. 4). As expected, the performances drop significantly, due to the reduced amount of data. Nevertheless, TSpred demonstrates higher ROC-AUC and PR-AUC compared to other methods, achieving a mean ROC-AUC of 0.553 and a mean PR-AUC of 0.397. When evaluated using the classification metrics, our model shows good accuracy, precision, and specificity (Supplementary Fig. S5). Overall, these results demonstrate the robustness of TSpred, and show that TSpred is least influenced by the bias caused by peptide imbalance in the dataset.

### 3.3 Prediction on unseen epitopes

**Figure 5.**
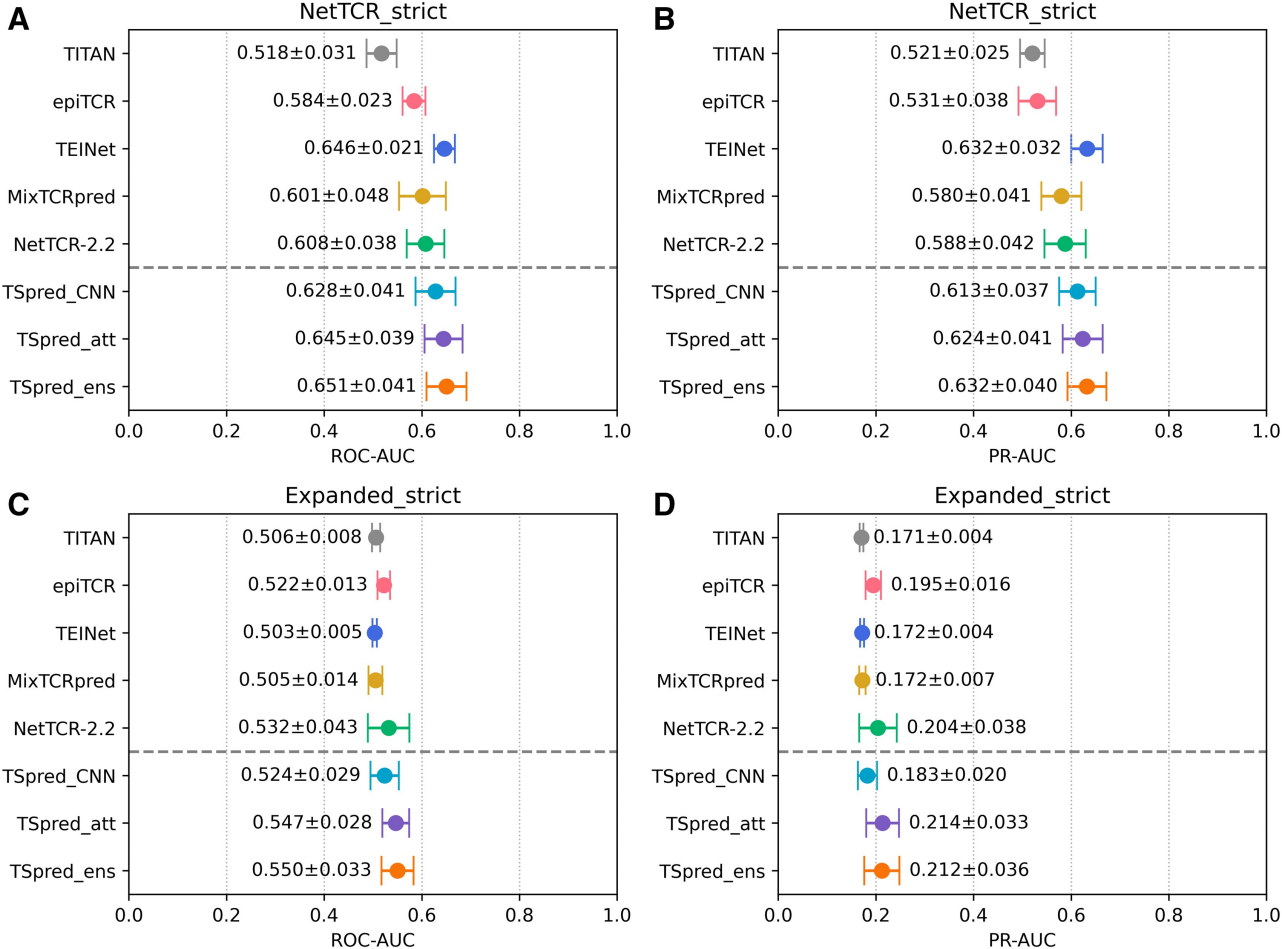
Performances of TSpred and other models on the unseen epitope datasets (NetTCR_strict, Expanded_strict). The colored dots represent the mean and the whiskers represent the standard deviation. (A) and (B) show the model performances in terms of ROC-AUC and PR-AUC on the NetTCR_strict dataset, respectively. (C) and (D) show the model performances in terms of ROC-AUC and PR-AUC on the Expanded_strict dataset, respectively. The upper part of each subplot shows the results of the compared state-of-the-art methods, whereas the lower part of each subplot shows the results of the final TSpred model (TSpred_ensemble) and its individual components (TSpred_CNN and TSpred_attention). TSpred_att stands for TSpred_attention; TSpred_ens stands for TSpred_ensemble.

We next move onto the task of specificity prediction for unseen epitopes, which is a much more challenging problem. We first analyze the model performances on the NetTCR_strict dataset, obtained by averaging the results from the five different cross validation runs (Fig. 5A and B). We find that TSpred_ensemble outperforms all other methods in terms of mean ROC-AUC, achieving a score of 0.651. In terms of mean PR-AUC, TSpred_ensemble is competitive with TEINet, achieving a score of 0.632. In terms of classification metrics, our model demonstrates good accuracy, precision, and specificity (Supplementary Fig. S6). We also analyze the model performances on the Expanded_strict dataset (Fig. 5C and D). Due to the greater difficulty of the dataset generated by random shuffling, models tend to show performances close to random. Still, TSpred_ensemble outperforms all other predictors, achieving a mean ROC-AUC of 0.550 and a mean PR-AUC of 0.212. In terms of classification metrics, our model demonstrates good accuracy, precision, and specificity (Supplementary Fig. S7). On both datasets, TSpred_attention demonstrates slightly better performances compared to TSpred_CNN, suggesting that the reciprocal attention mechanism helps the model to better capture the general features associated with TCR–epitope interactions.

Interestingly, TEINet demonstrates remarkably high performance on the NetTCR_strict dataset, contrasting with its poor performances on other datasets using the random shuffling strategy. Although it uses only beta chain data, it outperforms the methods using paired chain data. TEINet architecture incorporates two pre-trained encoders, one trained on 100 000 CDRβ3 sequences and the other trained on >300 000 epitope sequences (Jiang *et al.* 2023). The use of these pretrained encoders may lead to the ability of TEINet to easily discriminate between the positive TCRs and negative control TCRs, leading to its enhanced performance on the NetTCR_strict dataset. In addition, while TITAN was reported to achieve good performances on unseen peptides, it performs poorly when tested on our datasets. Upon examination of the source code of TITAN, we found that the authors of TITAN did not use a validation set for hyperparameter selection and simply chose the number of epochs based on the test set ROC-AUC, which can result in the overestimation of model performances. We speculate that the lack of a validation set, as well as the use of only beta chain data, are the reasons why TITAN did not perform well on unseen epitopes in our assessment.

Using the Expanded_strict dataset, we also analyze the relationship between the ROC-AUC performances for each test set peptide and the similarity between the training and test set peptides. To do so, we compute $L_{\min}$ of each test set peptide, which is the minimum Levenshtein distance of that peptide to the training set peptides. Levenshtein distance is a measure of the difference between two sequences, computed as the minimum number of single-character edits necessary to transform one sequence into another (Levenshtein 1966). We compute the average ROC-AUC of the peptides with maximum $L_{\min}$ to that of the peptides with minimum $L_{\min}$ for each test peptide from all 5-folds. The average ROC-AUC of the peptides with maximum $L_{\min}$ is 0.449, and the average ROC-AUC of the peptides with minimum $L_{\min}$ is 0.692 (Supplementary Fig. S8). These results suggest that a higher similarity of the test set peptide to the training set peptides correlates with a higher predictive performance for that peptide.

### 3.4 Analysis of attention scores

The reciprocal attention layer in TSpred_attention model has been conceived to capture the interaction patterns underpinning TCR–epitope binding. In order to examine whether the reciprocal attention layer can capture the structurally interacting residue pairs, we examine our model using the TCR-peptide complex structure data obtained from the STCRDab database (downloaded Aug. 2022) (Leem *et al.* 2018). In particular, we create a new dataset named ‘structure set’ composed of 24 STCRDab structures that contain peptides overlapping between NetTCR_full dataset and STCRDab (Supplementary Table S1). First, to train our model, we construct a training and validation set by randomly choosing 75% and 25% of the data from the total NetTCR_full dataset. Here, the CDRα and CDRβ sequences in TSpred_attention are generated by concatenating the CDR1, CDR2, and CDR3 sequences first, and then zero-padding the concatenated sequence. After training the model on NetTCR_full dataset, we obtain the attention scores assigned to peptide-CDRβ residue pairs for each structure in the structure set. Afterwards, we examine the relationship between the obtained attention scores and the pairwise distances of the peptide-CDRβ residue pairs. The attention scores and the pairwise distances for four selected PDB structures (3QDG, 5TEZ, 6VMX, 7N6E) are shown in Supplementary Fig. S9. As shown in the figures, the model tends to place higher attention scores on the residue pairs that are closely located in the 3D structure.

**Figure 6.**
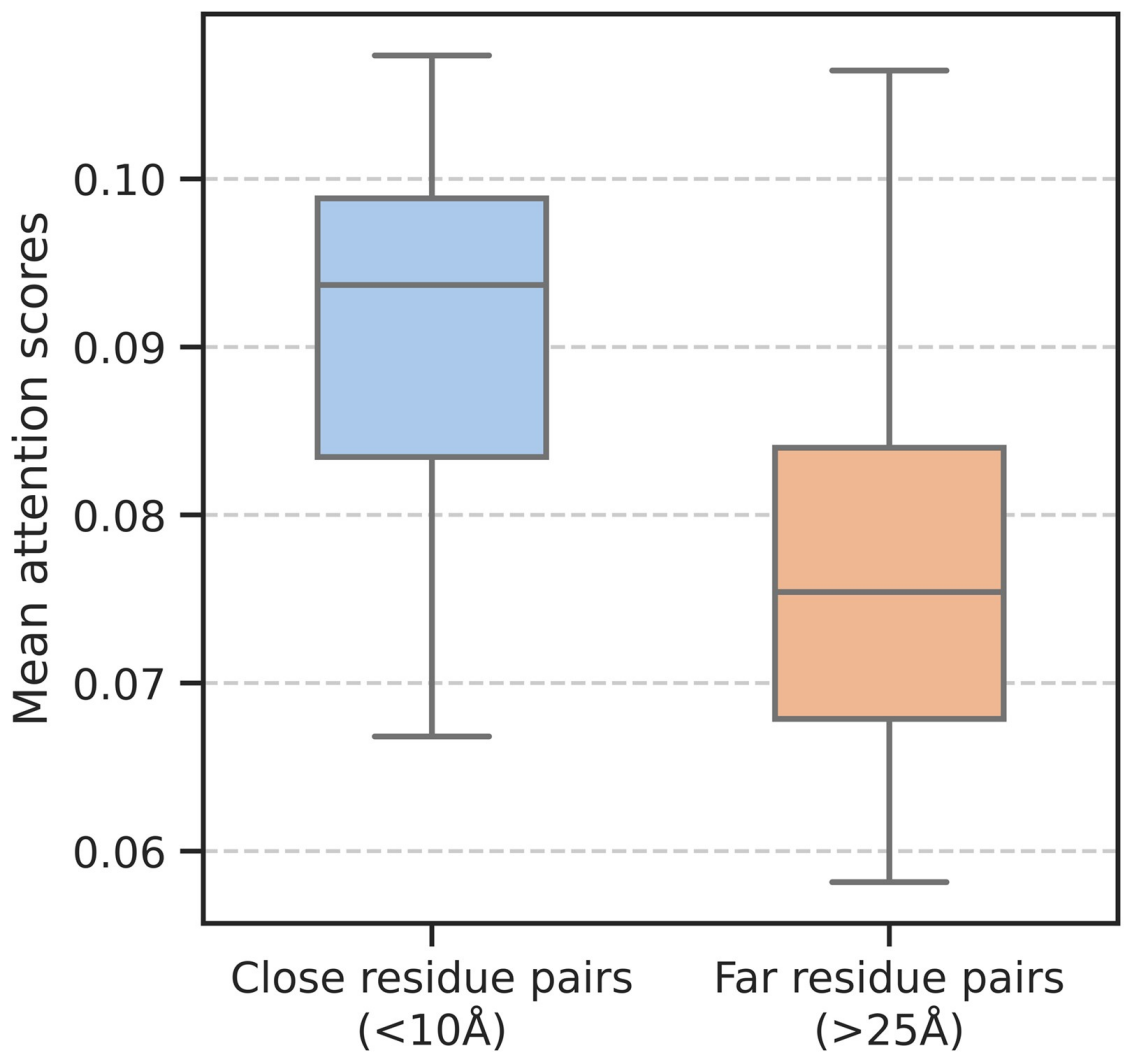
The mean attention score distributions for the close residue pairs (<10 Å) and the far residue pairs (>25 Å) for the 24 structures in the structure set.

The pairwise distances of all peptide-CDRβ residue pairs in the structure set have a mean of 16.45 Å and a standard deviation of 5.5 Å (Supplementary Fig. S10). Considering this, we assign a residue pair as a *close* residue pair if the distance is smaller than 10 Å, and a *far* residue pair if the distance is >25 Å. We compute the mean attention score for both the close and far residue pairs for each structure in the structure set. Then, we compare the distributions of scores for the close residue pairs and the far residue pairs. The scores of the close residue pairs show a mean and standard deviation of 0.091 ± 0.011, while the scores of the far residue pairs show a mean and standard deviation of 0.078 ± 0.013 (Fig. 6). The *t*-test testing for statistical significance gives a *P*-value of 3.15e−04, which implies that there is a significant difference between the two score distributions at the 99% confidence level. This indicates that the model generally places higher attention on the residue pairs that are located in close proximity. We also visualized a scatterplot of attention scores against the distances for all peptide-CDRβ residue pairs in the structure set (Supplementary Fig. S11), and observed a less obvious correlation.

## 4 Discussion

In this work, we propose TSpred, a new ensemble framework combining a CNN architecture with the attention mechanism for the prediction of TCR binding specificity. We take advantage of the unique strengths of each model: the strong ability of CNNs in feature extraction and pattern recognition, and the ability of the attention-based model to focus on the more important regions of the input. By formulating an integrated ensemble network, we are able to construct a reliable pan-specific prediction method by harnessing the predictive power of both models in learning TCR–epitope interactions. Upon a comprehensive assessment of our model, we find that TSpred achieves state-of-the-art performances on the prediction task for seen epitopes as well as unseen epitopes. In particular, we perform a robust evaluation of the model performance on the unseen epitope prediction task by designing two different datasets with different negative sampling strategies. The performances of TSpred_ensemble on these two datasets indicate its higher generalizability to unseen epitopes compared to the other predictors.

In our analysis on the seen and unseen epitope datasets, we observe that using paired chain data generally leads to an increase in model performance compared to using beta chain alone, confirming the results from a previous study (Meysman *et al.* 2023). Furthermore, one of the long-standing challenges in this field is the bias caused by peptide imbalance in the training data (Deng *et al.* 2023). Although it is a critical factor that affects all predictive methods including ours, TSpred demonstrates the most robust results among all compared predictors when assessed on a balanced dataset. Furthermore, the analysis of the attention scores shows that the model places higher attention on the residue pairs that are closely located in 3D space. Our model offers interpretability by showing which residue pairs are structurally interacting and thus important to TCR–epitope binding.

Each component of our model outperforms other previously published models based on similar architectures. For example, the pan-specific version of NetTCR-2.2 has a similar architecture compared to TSpred_CNN, but the two models differ in details such as the number of convolution layers and the kernel sizes. In model evaluation, TSpred_CNN outperforms NetTCR-2.2 in most of the test datasets. In addition, while TITAN and MixTCRpred are also methods based on the attention mechanism, TSpred_attention consistently outperforms these models. We reason that the model architecture design as well as the choice of hyperparameters are the contributing factors to the success of TSpred.

While the model proposed in this work demonstrates state-of-the-art performances on the prediction of TCR binding specificity, there are some notable limitations. The authors of NetTCR-2.2 kept peptides with at least 30 unique binding TCRs, resulting in 26 peptides. This procedure was done because the pairs corresponding to the low frequency epitopes may be noisy data, not representative of the true binding landscape. Although this may raise concerns about the limited number of epitopes, other works also have performed a similar procedure. The authors of NetTCR-2.1 (Montemurro *et al.* 2022) and IMMREP22 (Meysman *et al.* 2023) used stricter thresholds of 100 and 50 TCRs, respectively. Other studies such as TITAN (Weber *et al.* 2021), MixTCRpred (Croce *et al.* 2024), and TEINet (Jiang *et al.* 2023) used more lenient thresholds of 15, 10, and 10 TCRs, respectively. Considering the various thresholds used by different studies, we reason that the NetTCR_full dataset is acceptable. We expect that if more experimental data is accumulated in the future, it would be possible to overcome this issue. In addition, the dataset used in this work is restricted to paired chain data, which limits the amount of data available for model training. Considering that the publicly available data contain a large number of single chain information for either alpha or beta chain, it will be useful to develop a more advanced method that can handle both single and paired chain information. Another limitation is that the current model does not take into account the MHC allele information. We expect that incorporating the MHC allele information in future models will possibly lead to a better prediction accuracy.

Despite much efforts, the performances of currently developed models for unseen epitopes are still unsatisfactory for use in real clinical applications. In order to increase the reliability of prediction models in the future, we will need a greater amount of high-quality data. Currently available single-cell TCR sequencing data are reported to contain a considerable amount of noise (Povlsen *et al.* 2023). We expect that the advancement of denoising methods such as iTRAP (Povlsen *et al.* 2023) will be important in the future. Furthermore, numerous prediction tools are emerging in this field, employing diverse datasets, negative sampling strategies, and evaluation methodologies. As pointed out in the IMMREP benchmark study (Meysman *et al.* 2023), there is a need for an independent benchmark for a thorough evaluation of different methods.

Another possible direction of the research in this field is the development of models based on structural information. Currently, experimental structures of TCR-pMHC complexes are still lacking. However, the latest version of AlphaFold (Jumper *et al.* 2021) has reportedly achieved a considerable progress in the structure prediction of antibody-antigen complexes, which share many similarities with TCR-pMHC complexes. We anticipate that such progress will lead to better predictions of TCR-pMHC structures, which will facilitate the development of more reliable methods for the prediction of TCR binding specificity.

## Supplementary Material

btae472_Supplementary_Data

## Data Availability

The data underlying this article are available at https://github.com/ha01994/tspred.
