# Supplementary material for: TSpred: a robust prediction framework for TCR–epitope interactions using paired chain TCR sequence data
Source: Bioinformatics. 2024 Jul 25;40(8):btae472. doi: 10.1093/bioinformatics/btae472 (PMC11297499; doi:10.1093/bioinformatics/btae472)
Supplement: btae472_Supplementary_Data [file btae472_supplementary_data.docx]

**Supplementary Information for:**

**TSpred: a robust prediction framework for TCR-epitope interactions using paired chain TCR sequence data**

Ha Young Kim^1^, Sungsik Kim^2^, Woong-Yang Park^2,3,4^, Dongsup Kim^1,*^

^1^Department of Bio and Brain Engineering, Korea Advanced Institute of Science and Technology, Daejeon 34141, South Korea

^2^GENINUS Inc., Seoul, South Korea

^3^Samsung Genome Institute, Samsung Medical Center, Seoul, South Korea

^4^Department of Molecular Cell Biology, Sungkyunkwan University School of Medicine, Suwon, South Korea

*Corresponding author (kds@kaist.ac.kr)

**Supplementary Note S1. Model details and training hyperparameters**

The maximum sequence lengths of CDRα1, CDRα2, CDRα3, CDRβ1, CDRβ2, CDRβ3 are 7, 8, 22 and 6, 7, 23 respectively. The maximum peptide sequence length is 12. The one-hot encoding of the input sequences has a dimension of 21, including 20 amino acids and 1 for padding.

In the CNN-based model, the 1D convolutional layer has an input channel size of 21, an output channel size of 32, a kernel size of 2, and a stride of 1. The max pooling layer has a kernel size of 2 and a stride of 1. The fully-connected layer after the pooling layer outputs vectors of dimension 64.

In the attention-based model, the sequence embedding layer has an output dimension of 128. All attention layers (self-attention and reciprocal attention layers) are composed of a multi-head attention layer followed by dropout, residual connection, and layer normalization. For the multi-head attention layer, the number of heads is 4, the hidden dimension is 128, the key dimension is 32, and the value dimension is 32.

For both CNN- and attention-based models, we use a batch size of 128. For the CNN-based model, we use a learning rate of 1e-3 and a dropout rate of 0.6, and train for 200 epochs. For the attention-based model, we use a learning rate of 1e-4 and a dropout rate of 0.6, and train for 200 epochs.

**Supplementary Figure S1. Modified nested five-fold cross validation.** Unlike standard nested five-fold cross validation, which performs four folds in the inner loop and five folds in the outer loop, we simply use one fold in the inner loop and five folds in the outer loop, as shown below. In the inner loop, hyperparameter tuning is performed using the validation set. In the outer loop, model evaluation is performed on the test set.


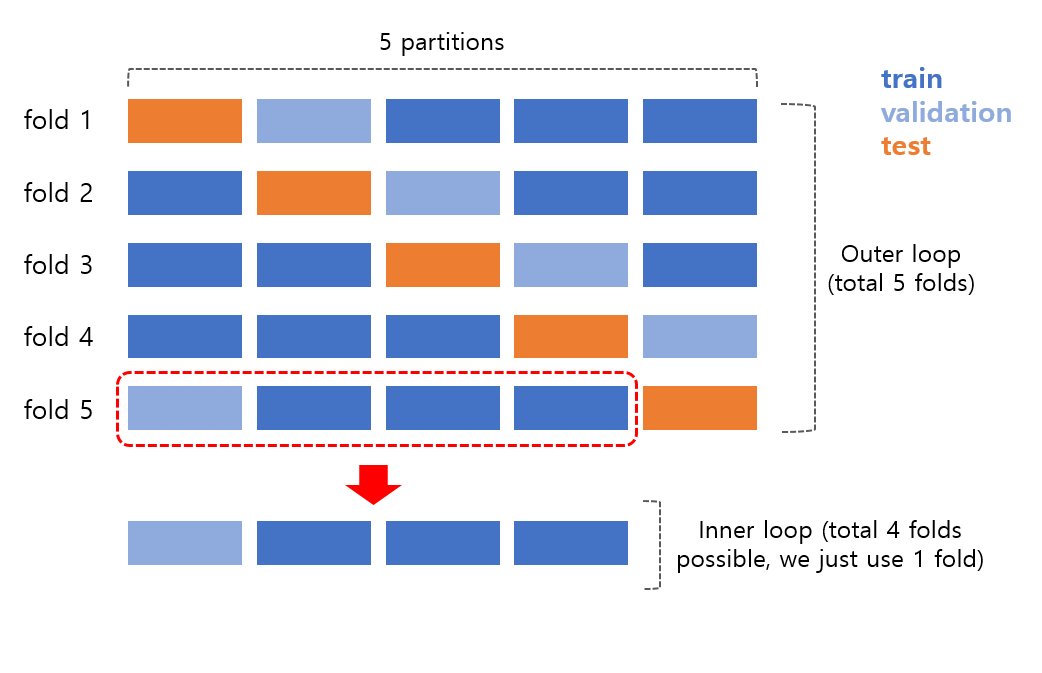


**Supplementary Figure S2. Illustration of the strict split scenario when there are not many epitopes in each fold.** A total of 15 epitopes are used in this illustration. Each partition contains 3 epitopes. The different colors indicate the samples corresponding to different epitopes. If there are too few epitopes in each fold, it is difficult to use random shuffling (which has to be done within each partition). For example, for the blue epitope in Partition 2, there are not enough data to sample negatives from.


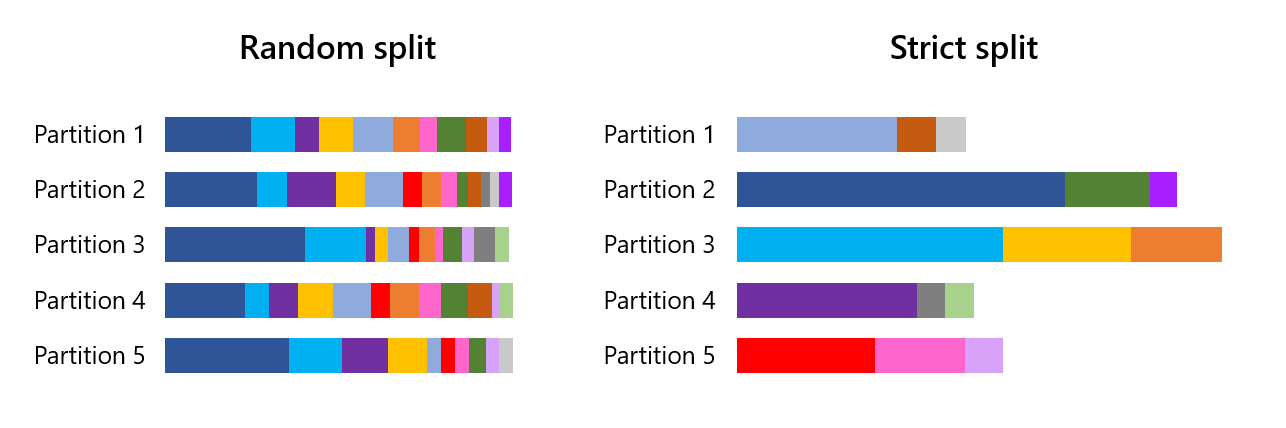


**Supplementary Figure S3. Classification results on the NetTCR_full dataset**


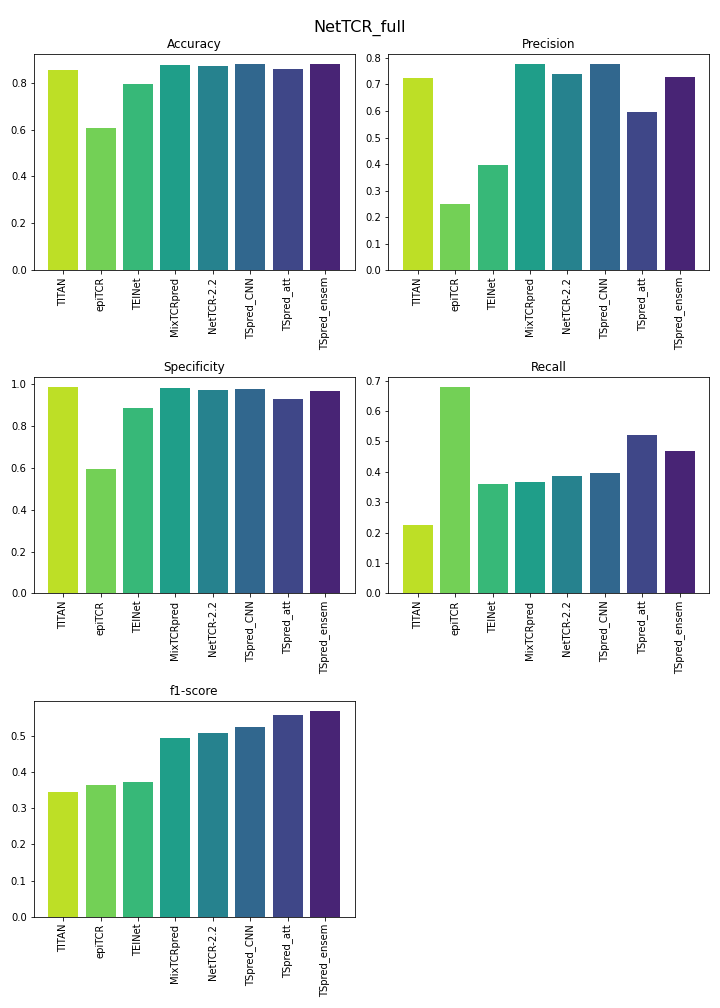


**Supplementary Figure S4. Classification results on the IMMREP dataset**


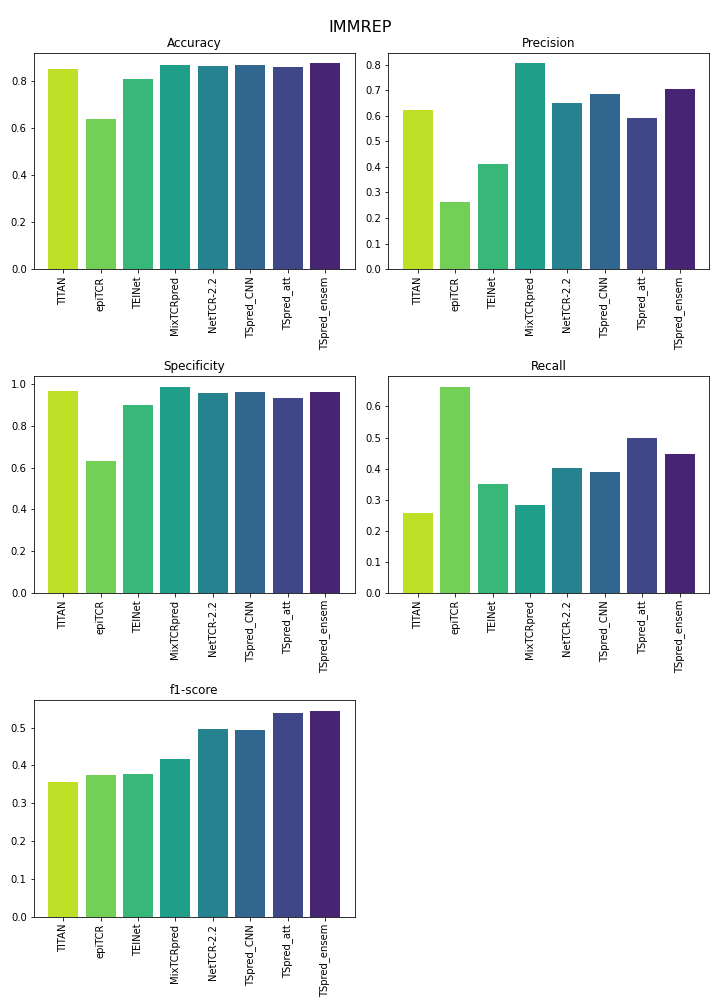


**Supplementary Figure S5. Classification results on the NetTCR_bal dataset**


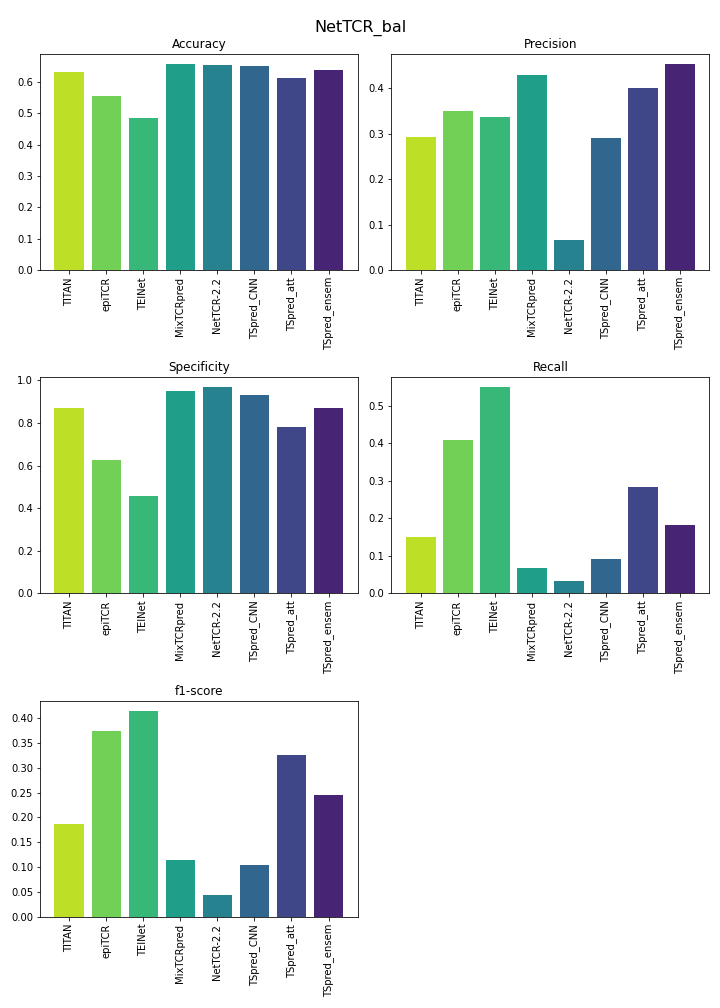


**Supplementary Figure S6. Classification results on the NetTCR_strict dataset**

**
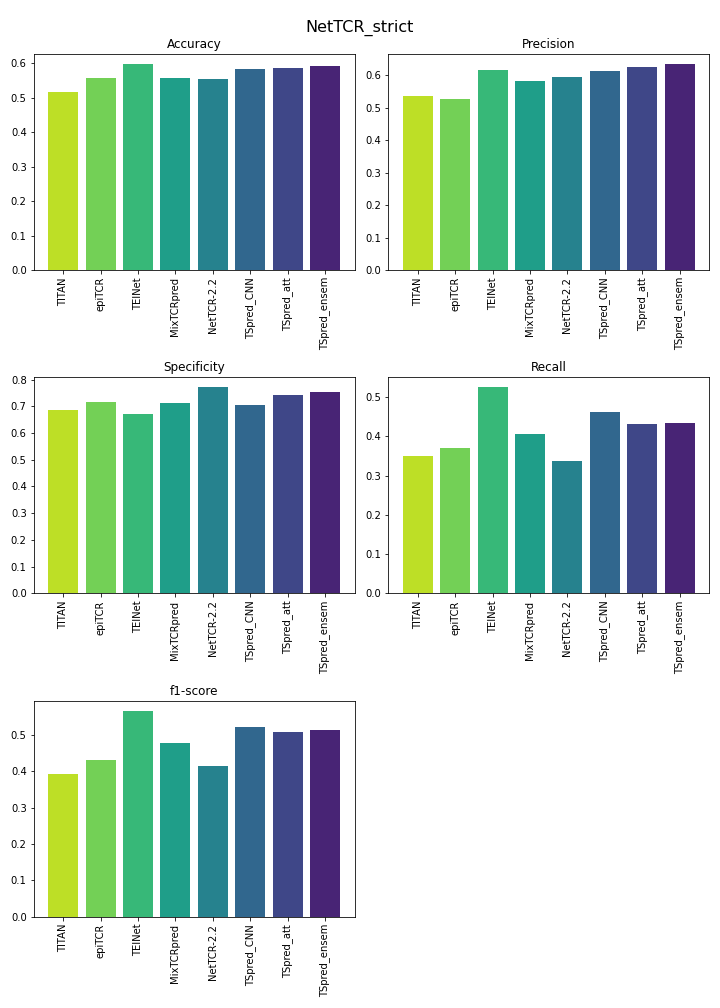
**

**Supplementary Figure S7. Classification results on the Expanded_strict dataset**

**
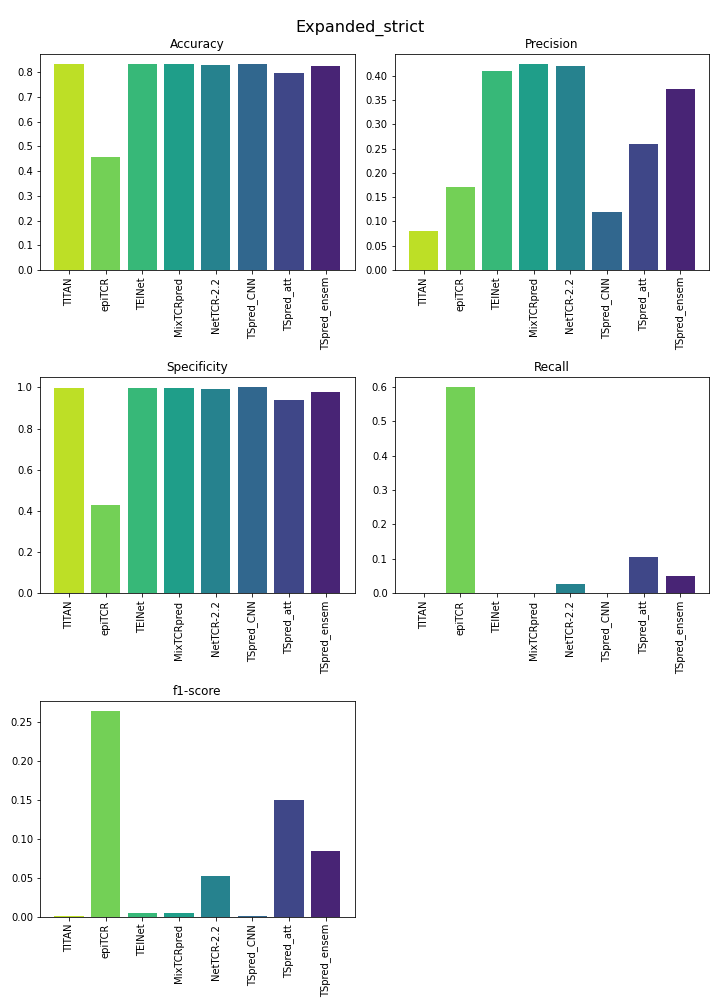
**

**Supplementary Figure S8. ROC-AUC per peptide for test set peptides in the Expanded_strict dataset with either maximum L_min (7) or minimum L_min (1).**


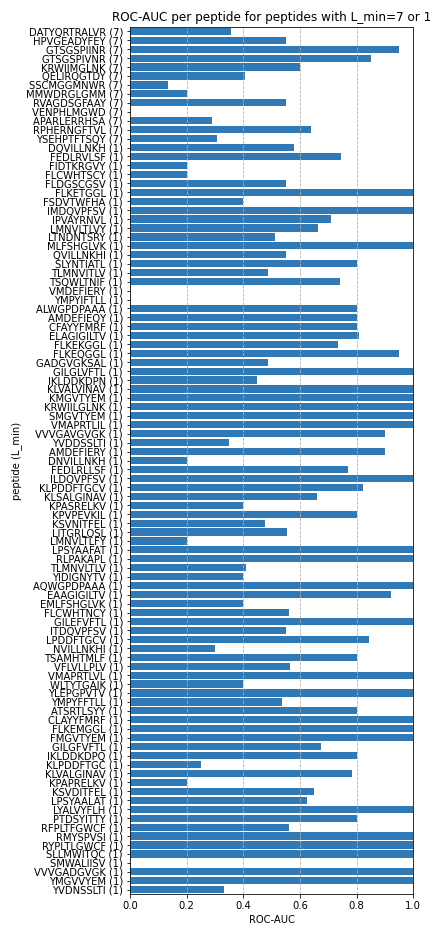


**Supplementary Figure S9. Pairwise residue distances and pairwise attention scores of four selected structures (3QDG, 5TEZ, 6VMX, 7N6E) in the structure set.** The x-axis labels show the CDR beta sequence (concatenation of the three CDRs from the beta chain) residues, while the y-axis labels show the peptide sequence residues. Distances are shown in Angstroms (Å).


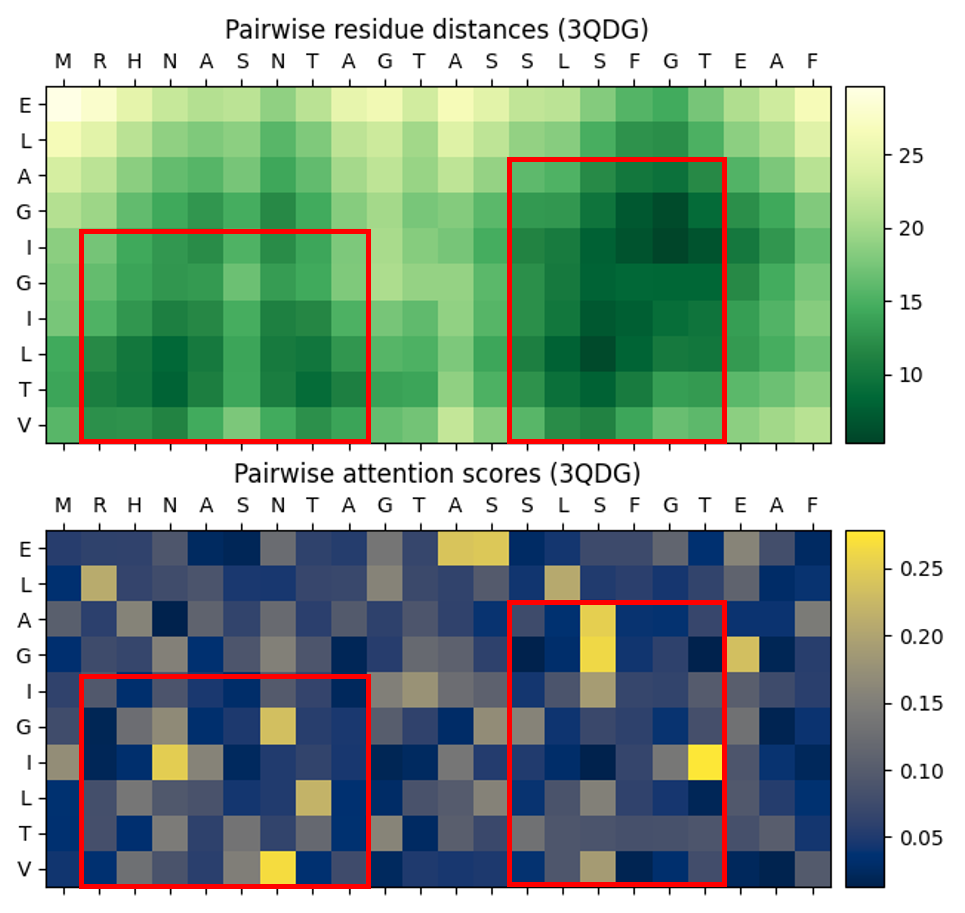


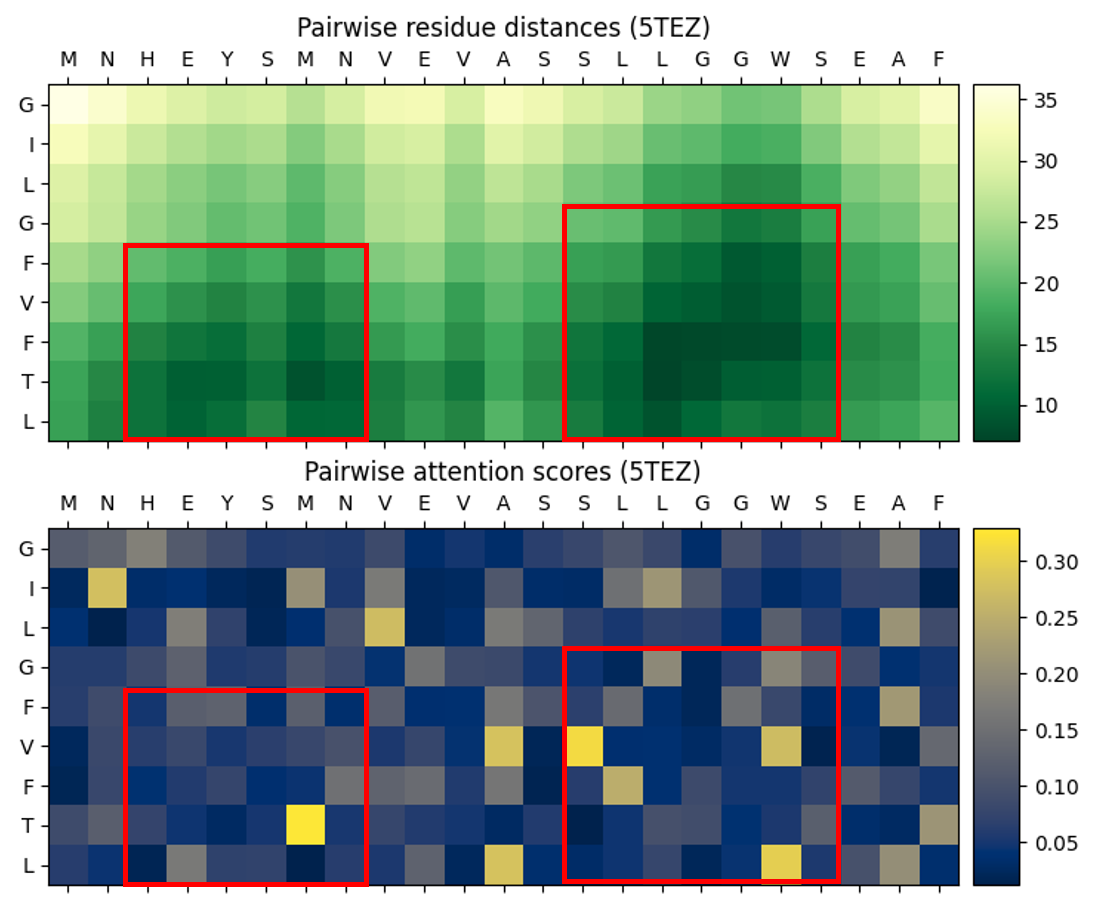


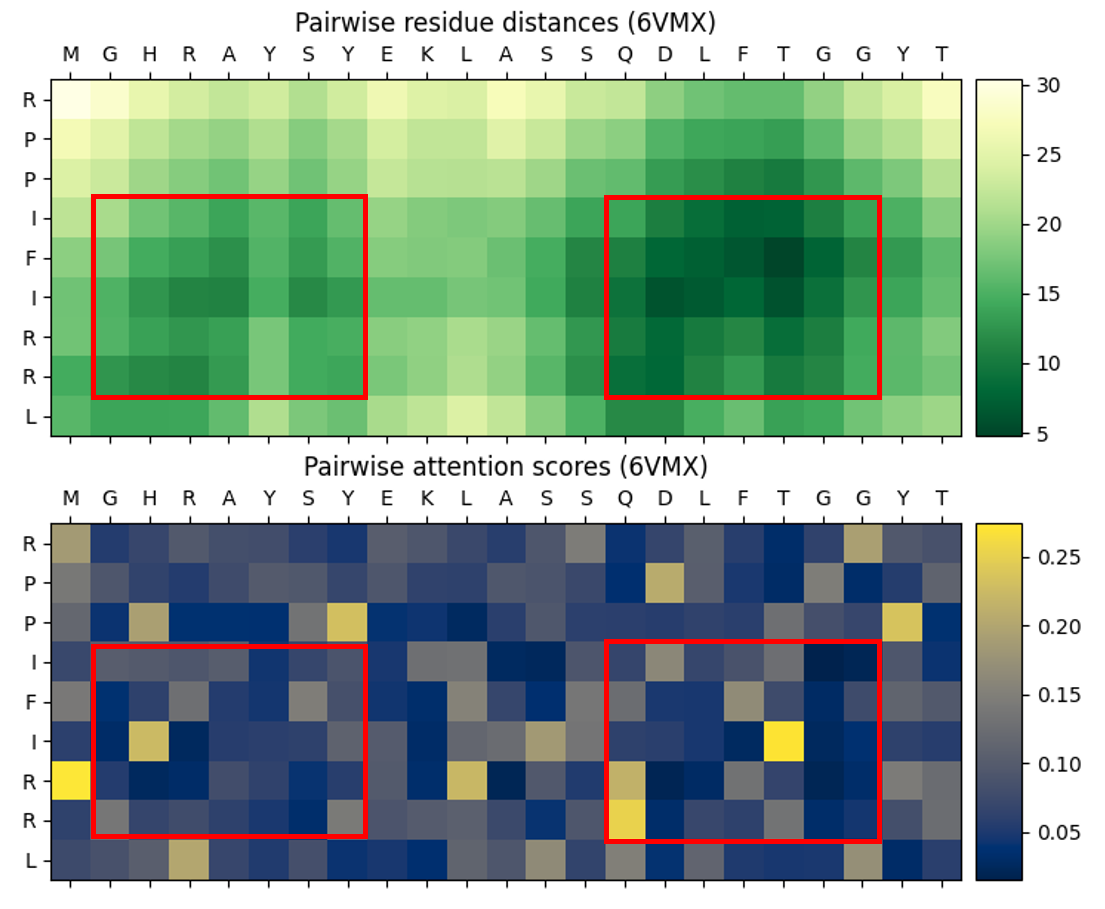


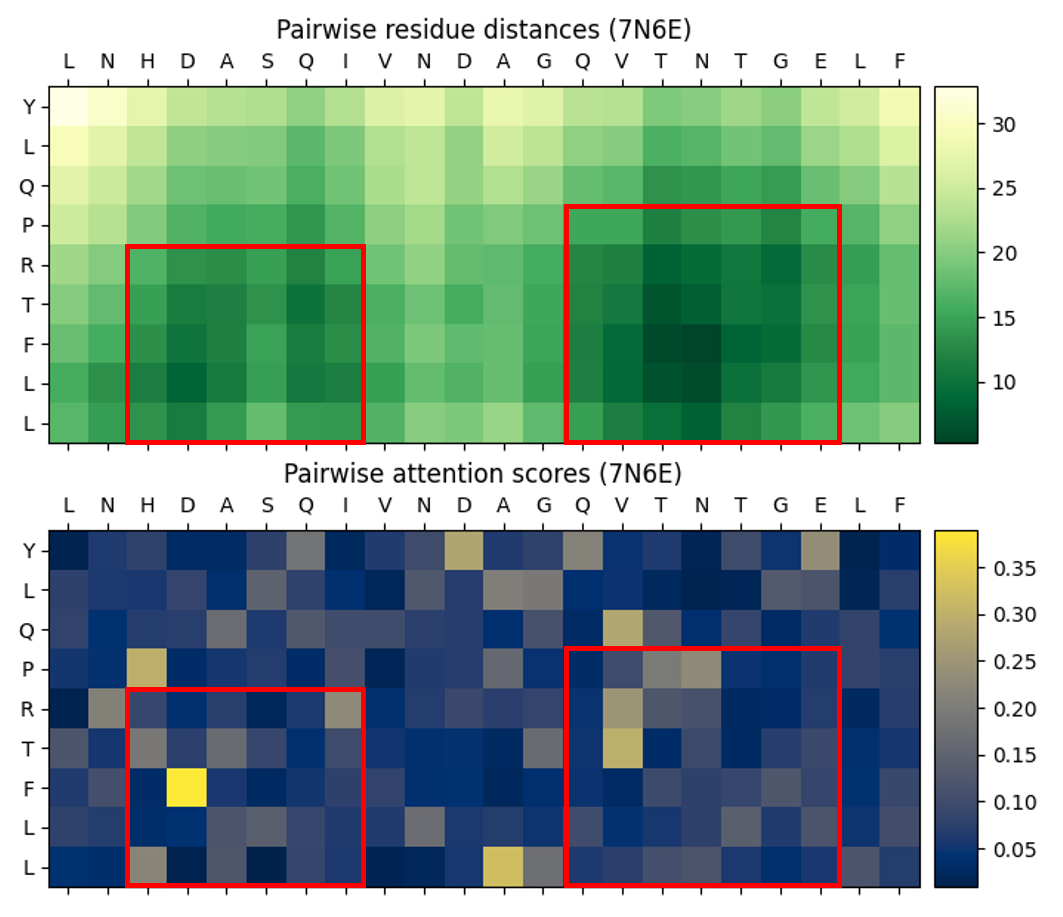


**Supplementary Figure S10. The distribution of the pairwise residue distances of all peptide-CDRβ residue pairs in the structure set.** The pairwise distances of all peptide-CDRβ residue pairs in the structure set have a mean of 16.45Å and a standard deviation of 5.5Å.


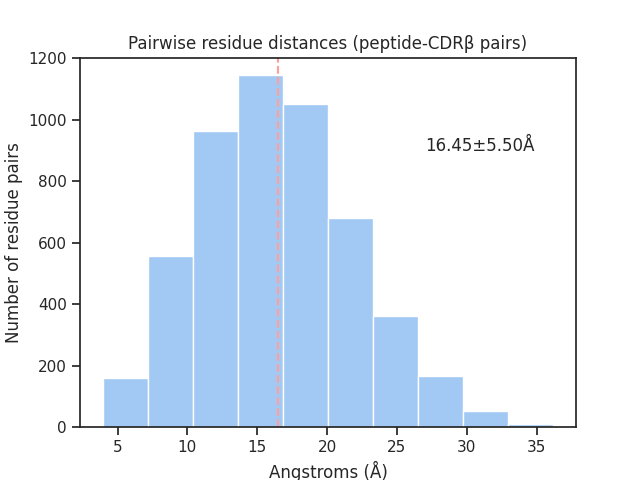


**Supplementary Figure S11. Scatterplot of attention scores against the distances for all peptide-CDRβ residue pairs in the structure set.** The computed Pearson correlation coefficient is -0.036.

**
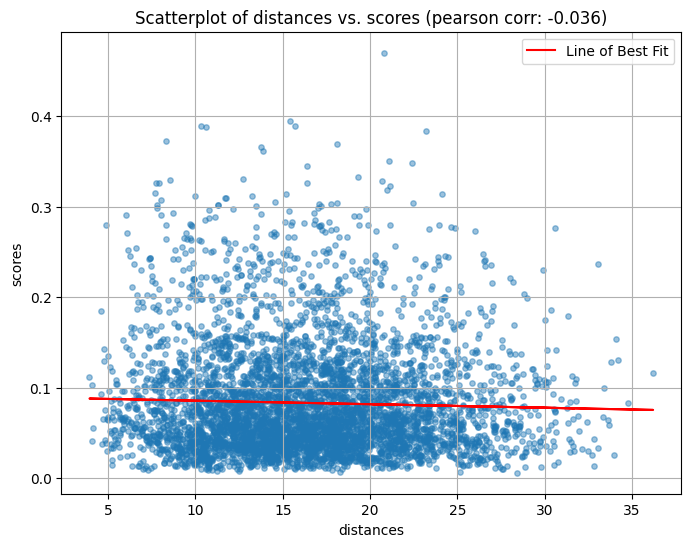
**

**Supplementary Table S1. Information of the ‘structure set’ composed of 24 STCRDab structures.**

| **PDB code** | **peptide** |
| --- | --- |
| 2VLK | GILGFVFTL |
| 2VLR | GILGFVFTL |
| 3GSN | NLVPMVATV |
| 3HG1 | ELAGIGILTV |
| 3O4L | GLCTLVAML |
| 3QDG | ELAGIGILTV |
| 3QDM | ELAGIGILTV |
| 3VXM | RFPLTFGWCF |
| 3W0W | RFPLTFGWCF |
| 4JFF | ELAGIGILTV |
| 4L3E | ELAGIGILTV |
| 5E9D | ELAGIGILTV |
| 5EUO | GILGFVFTL |
| 5ISZ | GILGFVFTL |
| 5JHD | GILGFVFTL |
| 5NHT | ELAGIGILTV |
| 5NQK | ELAGIGILTV |
| 5TEZ | GILGFVFTL |
| 6DKP | ELAGIGILTV |
| 6MTM | FEDLRVLSF |
| 6VMX | RPPIFIRRL |
| 7N6E | YLQPRTFLL |
| 7PBE | YLQPRTFLL |
| 7RTR | YLQPRTFLL |
